# Cardiopulmonary resuscitation in donation after brain death donors aged ≥65 years: impact on outcomes after kidney transplantation – a multi-center study

**DOI:** 10.3389/ti.2026.16028

**Published:** 2026-05-22

**Authors:** Can C. Süsal, Quirin Bachmann, Florian Kälble, Christoph F. Mahler, Matthias Ott, Johannes Heymer, Matthias Braunisch, Volker Assfalg, Jürgen Dippon, Uwe Heemann, Lutz Renders, Vedat Schwenger, Fabian Echterdiek

**Affiliations:** 1 Department of Nephrology, Klinikum Stuttgart - Katharinenhospital, Stuttgart, Germany; 2 Department of Nephrology, TUM Universitätsklinikum Rechts der Isar, Munich TUM School of Medicine and Health, Munich, Germany; 3 Department of Nephrology, Heidelberg University Hospital, Heidelberg, Germany; 4 Interdisciplinary Intensive Care Unit, ZIM, Klinikum Stuttgart - Katharinenhospital, Stuttgart, Germany; 5 Department of Surgery, TUM Universitätsklinikum Rechts der Isar, Munich, TUM School of Medicine and Health, Munich, Germany; 6 Institute for Stochastics and Applications, University of Stuttgart, Stuttgart, Germany

**Keywords:** cardiopulmonary resuscitation, donation after brain death, elderly donors, kidney transplant, transplant outcome

## Abstract

A history of cardiopulmonary resuscitation (CPR) is common in donation after brain death (DBD) donors. While good outcomes have been demonstrated for kidney transplantation (KT) from younger CPR donors (aged typically 18–50 years), it is unclear whether this is true for the growing cohort of ≥65-year-old KT donors. To this end, all KTs from ≥65-year-old DBD donors performed at three German transplant centers from January 2006 to December 2023 (n = 680) were retrospectively analyzed and outcomes of KTs from donors with and without a history of CPR were compared (n = 81 and n = 599, respectively). No significant differences were observed regarding the incidence of delayed graft function (DGF) as well as regarding 1- and 5-year graft function between the CPR and no-CPR groups (DGF: 27.2% vs. 33.1%, p = 0.40; 1-year eGFR (mL/min): 33.3 vs. 35.0, p = 0.75; 5-year eGFR: 35.8 vs. 37.3, p = 0.75, respectively). Death-censored graft survival (73.8% vs. 66.0%, p = 0.24) and patient survival (78.7% vs. 73.5% p = 0.61) were comparable after 5 years between the CPR and no-CPR groups. The results were confirmed by multivariable Cox regression analysis. In conclusion, our results indicate that ≥65-year-old DBD donors with a history of CPR are potentially suitable for KT without impairing allograft outcomes.

## Graphical Abstract

**Figure:**
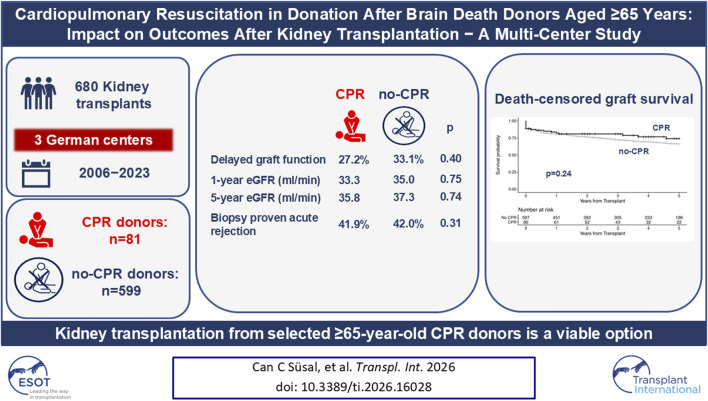

## Introduction

Kidney transplantation (KT) remains the best available therapeutic option for patients suffering from end-stage renal disease. The universal scarcity of deceased donor organs as well as the demographic change have resulted in a continuous increase in the proportion of elderly donors and donors with comorbidities over the last decades [1]. Nowadays, more than 40% of donors in the Eurotransplant area are expanded criteria donors (ECD) and more than 25% of donors are aged ≥65 years [2–4]. Despite these developments, waiting times remain a challenge so that further measures to expand the donor pool are necessary.

Cardiopulmonary resuscitation (CPR) is a major reason for admission to intensive care units and - despite best efforts - it is often followed by irreversible brain damage and brain death. Several studies have demonstrated that kidneys from younger donors (aged typically 18–50 years) with a history of CPR can be safely transplanted without compromising long-term allograft survival [5, 6]. Whether the same is true for the particularly vulnerable but growing group of ≥65-year-old donors is largely unknown even though nowadays transplant centers regularly receive such offers. A recent, single-center analysis from our group had suggested no negative effect of a history of CPR on allograft survival and function in KTs from ≥65-year-old donors [7]. However, additional data are needed to better guide management decisions in this challenging group of high-risk CPR donors. To this end, we performed a multi-center retrospective study to analyze outcomes of KTs from ≥65-year-old donation after brain death (DBD) donors with and without a history of CPR.

## Materials and methods

### Patient population

For this study, all KTs from DBD donors aged ≥65 years that were performed at three German transplant centers (Universitätsklinikum Heidelberg, Klinikum Stuttgart, and TUM Universitätsklinikum Rechts der Isar, Munich) between 01/01/2006 and 31/12/2023 were analyzed retrospectively (n = 739). The three centers have similar acceptance policies in that donors with an active history of malignancy or donors with certain untreated infections (e.g., HIV) are not accepted. Other than that, every offer is assessed on a case-by-case basis without predefined exclusion criteria.

Information on donor CPR was available from Eurotransplant in 680 of the 739 cases (92.0%) and only those KTs were included in this analysis. The number of KTs (n = 680) was higher than the number of kidney donors (n = 569) as we accepted both kidneys from the same donor and transplanted them into two separate recipients in 19.5% of all cases. In these cases, both KTs were included in this analysis. There were no double kidney transplantations (i.e., two kidneys from the same donor into one recipient). All KTs were from DBD donors since donation after circulatory death (DCD) is not allowed in Germany. In consequence, all donors with a history of CPR had undergone successful resuscitation, either in- or out-of-hospital, and were subsequently declared braindead during the following ICU stay. Combined organ transplants were excluded. All donor kidneys were maintained in cold storage without machine perfusion.

To analyze possible differences in post-transplant outcomes depending on the duration of CPR, we further stratified the CPR group into a short and long CPR group with the median duration of CPR (20 min) as a cut-off. The short CPR group (CPR duration <20 min) consisted of 39 KTs from 35 donors and the long CPR group (CPR duration ≥20 min) consisted of 42 KTs from 33 donors.

Recipients received a baseline immunosuppression consisting of a calcineurin inhibitor (either tacrolimus or cyclosporine), mycophenolate mofetil and steroids. An interleukin-2 receptor antibody (basiliximab) was used as standard induction therapy whereas patients with increased immunological risk (e.g., re-transplantation) received anti-thymocyte globulin (ATG) at the physician’s discretion.

### Data collection

Donor-related data were obtained from the Eurotransplant Registry in Leiden, The Netherlands. Recipient- and transplant-related data as well as follow-up data were retrieved from the electronical patient records at Klinikum Stuttgart, Universitätsklinikum Heidelberg and TUM Universitätsklinikum Rechts der Isar, Munich and from the Eurotransplant Registry. All data were saved in an electronical database. As not all patients were followed at the three transplant centers for the whole post-transplant time, local nephrologists were contacted for follow-up data if necessary. The study was approved by the local ethics committees at Heidelberg (S-187/2022), Munich (2023-313-S-KH) and Tübingen (632/2019BO2) and performed according to the ethical standards of the Declaration of Helsinki.

### Outcome

The primary outcomes were 5-year death-censored graft as well as patient survival. Secondary outcome measures included incidence of delayed graft function (DGF), primary non-function (PNF), eGFR at 3 months, 1 year, 3 years and 5 years after KT as well as the incidence of biopsy proven acute rejection (BPAR) episodes within the first 3 years after KT. Recipient eGFR (ml/min/1.73 m^2^) was calculated using the chronic kidney disease epidemiology collaboration (CKD-EPI) formula [8]. DGF was defined as the need for one or more dialysis sessions during the first week after transplantation. BPAR was graded according to the respective Banff classification at the time of biopsy. All types of rejection (borderline rejection, T-cell mediated and antibody-mediated) were counted as BPAR. Acute kidney injury (AKI) stage was defined according to the 2012 KDIGO Clinical Practice Guideline for Acute Kidney Injury [9]. All AKI donors were additionally classified as either having “ongoing” AKI if the serum-creatinine value at organ recovery was equal to the peak creatinine value or as “resolving” AKI if the creatinine value at organ recovery was lower than the peak creatinine value.

### Statistical analysis

Numerical data were summarized as medians with interquartile ranges (IQRs), and categorical data as percentages. The Kruskal–Wallis test assessed differences in distributions of numerical variables, and Fisher’s exact test examined associations between categorical variables.

For right-censored longitudinal data, survival was estimated using the Kaplan−Meier method, with group comparisons by log-rank test. We used Cox proportional hazards models to assess risk factors, accounting for potential confounders and variables involved in donor-recipient matching and incorporating random effects for transplant centers and for kidneys from the same donor. Donor (age, sex, BMI, cause of death, diabetes, arterial hypertension), recipient (age, sex, BMI, dialysis duration, diabetes, arterial hypertension, highest panel reactive antigen, number of transplants), and transplant (HLA mismatches, cold ischemia time) variables were included as covariates. The proportional hazards assumption was checked. Missing data were handled by multiple imputation.

Analyses were conducted in R (version 4.4.2) [10] using packages for survival analysis [11], mixed-effects modelling [12], and multiple imputation [13].

## Results

A total of 680 KTs from 569 DBD donors were included in this study. Of these, 81 KTs (11.9%) were from 68 donors with a history of CPR prior to organ donation (CPR group). The remaining 599 KTs originated from 501 donors that were not resuscitated prior to transplantation (no-CPR group).

Donor demographics are shown in Table 1. Donor age was slightly lower in the CPR group compared to the no-CPR group (median 70.0 vs. 72.0 years, p = 0.02). Donors in the CPR group died more often from non-cerebrovascular and non-traumatic causes (primarily anoxic brain damage; 42.6%), whereas donors in the no-CPR group died mostly from intracranial bleeding (58.9%, p < 0.001). No significant differences were observed regarding a donor history of arterial hypertension, diabetes, or smoking.

**TABLE 1:** Donor characteristics of kidney transplants, stratified according to donor history of CPR.

Characteristic	CPR Group (n = 68)	No-CPR Group (n = 501)	p-value
Age (years), median (IQR)	70 (68.0, 73.0)	72 (68.0, 77.0)	0.02*
Male sex	28 (41.2%)	237 (47.3%)	0.37
BMI, median (IQR)	26.9 (24.7, 29.3)	26.2 (24.2, 28.7)	0.09
Arterial hypertension	47 (71.2%)	295 (63.3%)	0.22
Diabetes	14 (22.6%)	77 (17.0%)	0.29
Smoking	20 (30.8%)	108 (24.3%)	0.28
Cause of death	​	​	<0.001*
Stroke Intracranial bleeding Trauma Other (e.g., anoxic brain damage)	9 (13.2%) 28 (41.2%) 2 (2.9%) 29 (42.6%)	62 (12.4%) 295 (58.9%) 58 (11.6%) 86 (17.2%)	​
eGFR (ml/min/1.73 m^2^)	​	​	​
On admission, median (IQR) Lowest, median (IQR) Final, median (IQR)	66.0 (55.6, 77.0) 60.5 (39.0, 73.3) 67.6 (46.0, 91.4)	84.4 (68.3, 92.0) 71.3 (57.5, 87.4) 81.0 (62.3, 93.0)	<0.001* <0.001* 0.004*
Diuresis in last 24 h prior to organ donation (L), median (IQR)	3.0 (1.9, 4,0)	3.6 (2.4, 4.9)	0.03*
AKI stage	​	​	0.001*
No AKI Stage 1 Stage 2 + 3	37 (54.4%) 26 (38.2%) 5 (7.4%)	379 (76.6%) 100 (20.2%) 16 (3.2%)	​
Ongoing AKI at kidney recovery	13 (39.4%)	80 (64.0%)	0.02*
Time from admission to organ procurement (days), median (IQR)	4 (2, 5)	3 (2, 5)	0.79
KDPI, median (IQR)	78 (61.0, 89.0)	74.5 (62.0, 88.0)	0.82
Duration of CPR, median (IQR)	20 (12.5, 30)	​	​
Out-of-hospital cardiac arrest	58 (85.2%)	​	​

Percentages are based on the number of available cases for each parameter. Kruskal–Wallis test was used for comparison of numeric variables and Fisher´s exact test (i.e., Chi2-test) was used for comparison of categorical variables. AKI, acute kidney injury; BMI, body mass index; CPR, cardiopulmonary resuscitation; CVA, cerebrovascular accident; IQR, interquartile range; * if p < 0.05.

Donors with a history of CPR had significantly lower admission, minimum and final eGFR levels compared to the no-CPR group (on admission: 66.0 mL/min vs. 84.4 mL/min, p < 0.001; minimum: 60.5 mL/min vs. 71.3 mL/min, p < 0.001; final: 67.6 mL/min vs. 81.0 mL/min, p = 0.004). Consequently, a donor AKI was seen significantly more often in the CPR group (45.6% vs. 23.4%, p = 0.001). Diuresis in the last 24h prior to organ recovery was also slightly lower in the CPR group (3.0 L vs. 3.6 L, p = 0.03) but no donor in either group was oliguric (i.e., diuresis <500mL/24h), anuric or on hemodialysis. Interestingly, an ongoing AKI at organ recovery was present significantly more often in the no-CPR group than in the CPR group (64.0% vs. 39.4%, p = 0.02).

The median duration of CPR was 20 min with 80% of all CPRs lasting ≤30 min. The vast majority of CPRs occurred out-of-hospital (85%). Stratification of the CPR group according to the median CPR duration revealed that donors in the long CPR group were younger than donors in the short CPR group and the no-CPR group (70 vs. 72 vs. 72 years, p = 0.04). Also, a donor history of diabetes and smoking were each significantly more frequent in the long CPR group (Supplementary Table 1). The other results were similar as observed for the whole CPR group.

Recipient characteristics are displayed in Table 2. The median recipient age was 67.0 years in the CPR group and 67.0 years in the no-CPR group (p = 0.88). No significant differences were found regarding the duration of dialysis prior to KT, underlying renal disease, history of diabetes mellitus or arterial hypertension, number of HLA mismatches, cold ischemia time (CIT) and baseline immunosuppression. The results for the subgroup analysis stratified according to the duration of donor CPR revealed the same results and are displayed in Supplementary Table 2.

**TABLE 2:** Recipient and transplant characteristics of kidney transplants, stratified according to donor history of CPR.

Characteristic	CPR Group (n = 81)	No-CPR Group (n = 599)	p-value
Age (years), median (IQR)	67 (65.0, 69.0)	67 (65.0, 70.0)	0.88
Male sex	56 (69.1%)	404 (67.4%)	0.80
BMI, median (IQR)	26.9 (24.4, 29.2)	25.7 (23.6, 29.1)	0.14
Duration of dialysis (months), median (IQR)	50 (34, 73)	52 (31, 75)	0.91
RRT	​	​	0.36
Hemodialysis Peritoneal dialysis	69 (85.2%) 12 (14.8%)	530 (88.8%) 67 (11.2%)	​
Underlying renal disease	​	​	0.45
Diabetic nephropathy Hypertensive nephropathy Polycystic kidney disease Glomerulonephritis Other Unknown	10 (12.3%) 14 (17.3%) 11 (13.6%) 25 (30.9%) 12 (14.8%) 9 (11.1%)	72 (12.0%) 59 (9.9%) 83 (13.9%) 180 (30.1%) 121 (20.2%) 83 (13.9%)	​
Arterial hypertension	65 (89.0%)	445 (87.3%)	0.85
Diabetes	17 (23.3%)	119 (23.3%)	1.00
Highest PRA	​	​	<0.01*
0% >0 – ≤20% >20%	52 (64.2%) 13 (16.0%) 16 (19.8%)	337 (57.3%) 189 (32.1%) 62 (10.5%)	​
Second/third kidney transplant	11 (13.5%)	40 (6.7%)	0.07
HLA mismatches	​	​	0.33
0 1–2 3–4 5–6	3 (3.7%) 12 (14.8%) 36 (44.4%) 30 (37.0%)	10 (1.7%) 64 (10.8%) 283 (47.6%) 238 (40.0%)	​
Cold ischemia time (hours), median (IQR)	10.2 (8.2, 13.5)	11.2 (8.1, 15.7)	0.22
Immunosuppression	​	​	​
IL2-RA/ATG/none Tac/CsA/other MMF/Aza Corticosteroids	84.0%/14.8%/1.2% 70.4%/29.6%/0.0% 100%/0.0% 98.8%	88.7%/7.5%/3.8% 56.0%/43.1%/0.8% 99.7%/0.3% 98.0%	0.06 0.05 1 1
Year of transplantation, median (IQR)	2016 (2011, 2018)	2014 (2010, 2018)	0.11

Percentages are based on the number of available cases for each parameter. Kruskal–Wallis test was used for comparison of numeric variables and Fisher´s exact test (i.e., Chi2-test) was used for comparison of categorical variables. AKI, acute kidney injury; IQR, interquartile range; BMI, body mass index; RRT, renal replacement therapy; PRA, panel reactive antigen; HLA, human leukocyte antigen; SD, standard deviation, IL2-RA, interleukin 2-receptor antibody; ATG, antithymocyte globulin; Tac, tacrolimus; CsA, Ciclosporine A; MMF, mycophenolate mofetil; Aza, azathioprine, ESP, Eurotransplant Senior Program; * if p < 0.05.

**FIGURE 1:**
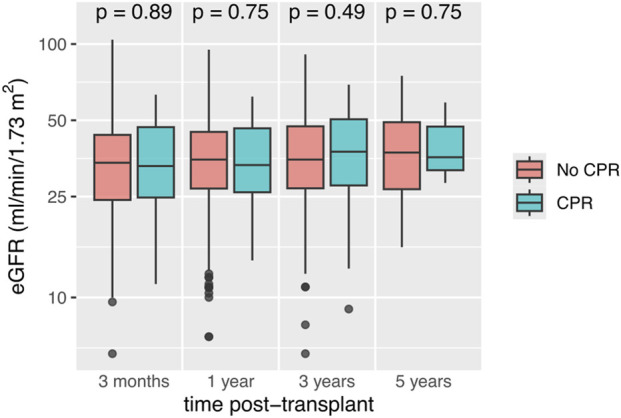
Recipient eGFR (based on CKD-EPI formula) up to 5 years after kidney transplantation from donors with and without a history of cardiopulmonary resuscitation (CPR). Box-and-whisker plots display the medians and interquartile ranges (IQR) of each sample. The whiskers extend to the smallest and largest values within 1.5 times the IQR. Data points beyond this range are marked with circles and represent outliers. The Kruskal–Wallis test was used to assess whether group distributions are equal. eGFR, estimated glomerular filtration rate.

The incidence of DGF was comparable between the two groups (CPR group: 27.2% vs. no-CPR group 33.1%, p = 0.40). PNF also occurred at similar rates (CPR group: 9.9% vs. no-CPR group 12.2%, p = 0.19). Both groups had similar median follow-up time (CPR group 37.5 months vs. no-CPR group 41.0 months; p = 0.30). During this follow-up time, graft function did not show significant differences at any assessed time point (3 months, 1 year, 3 years and 5 years) between the two groups (Table 3; Figure 1). The incidence of BPAR within the first three years post KT was also very similar (CPR group 41.9% vs. no-CPR group 42.0%, p = 0.31). The subgroup analysis stratified according to duration of CPR revealed essentially the same results (Supplementary Table 3). The DGF rate was 25.6% in the short CPR group, 28.6% in the long CPR group and 33.1% in the no-CPR group (p = 0.38). Graft function and incidence of BPAR were also not different between the short CPR group, the long CPR group, and the no-CPR group.

**TABLE 3:** Short and long-term outcomes of kidney transplants stratified by donor history of CPR.

Characteristic	CPR Group (n = 81)	No-CPR Group (n = 599)	p-value
DGF	22 (27.2%)	197 (33.1%)	0.40
PNF	8 (9.9%)	73 (12.2%)	0.19
Recipient eGFR (ml/min/1.73 m^2^), median (IQR)	​	​	​
3 months after transplant 1 year after transplant 3 years after transplant 5 years after transplant	33.0 (24.8, 47.0) 33.3 (26.0, 46.5) 37.6 (27.7, 50.5) 35.8 (31.8, 47.3)	34.0 (24.3, 43.8) 35.0 (26.9, 45.0) 35.0 (27.0, 47.4) 37.3 (26.8, 49.2)	0.89 0.75 0.49 0.74
BPAR in first three years after KT	31 (41.9%)	231 (42.0%)	0.31
Death-censored graft survival	​	​	0.24
1-year 3-year 5-year	82.1% 80.8% 73.8%	80.0% 72.1% 66.0%	​
Patient survival	​	​	0.61
1-year 3-year 5-year	89.9% 81.1% 78.7%	90.2% 80.2% 73.5%	​
Follow-up time (months), median (IQR)	37.5 (21.0, 60.2)	41.0 (23.0, 81.0)	0.30

Percentages are based on the number of available cases for each parameter. Kruskal–Wallis test was used for comparison of numeric variables and Fisher´s exact test (i.e., Chi2-test) was used for comparison of categorical variables. Death-censored graft survival and patient survival rates were calculated using the Kaplan−Meier method; AKI, acute kidney injury; BPAR, biopsy proven acute rejection; DGF, delayed graft function; PNF, primary non-function; IQR, interquartile range.

**FIGURE 2:**
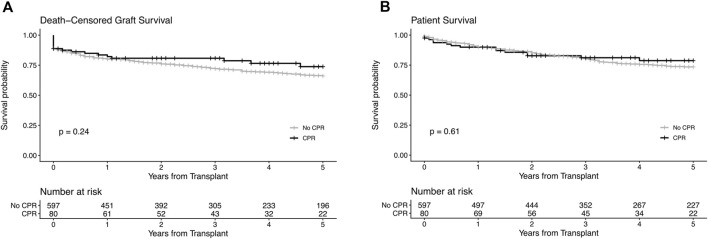
Kaplan−Meier estimates for death-censored graft survival **(A)** and patient survival **(B)** of kidney transplants from donors with and without a donor history of cardiopulmonary resuscitation (CPR). The log-rank test was used to assess whether group distributions are equal.

**FIGURE 3:**
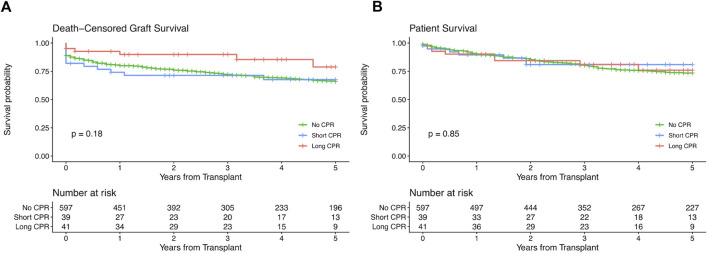
Kaplan−Meier estimates for death-censored graft survival **(A)** and patient survival **(B)** of kidney transplants stratified by duration of cardiopulmonary resuscitation (CPR). Short CPR means a CPR duration of up to 20 min and long CPR means a CPR duration of 20 min or longer. The log-rank test was used to assess whether group distributions are equal.

Five-year death-censored graft survival and patient survival were comparable between donors with and without a history of CPR (p = 0.24 and p = 0.61 respectively, see Figures 2A,B). After stratification into donors with short and long CPR we neither observed differences regarding 5-year death-censored graft nor patient survival between the groups (Figures 3A,B).

For further validation of our results, we subsequently performed a multivariable Cox regression analysis using a multiple imputation approach with adjustment for known risk factors and confounding factors of death-censored graft loss and death (Table 4). Random effects were included to capture the influence of the three transplant centers and to account for the non-independence of kidney transplants originating from the same donor. CPR did not increase the risk of death-censored graft loss (Hazard Ratio [HR] 0.76, 95% confidence interval [CI] 0.48–1.21, p = 0.25) or death (HR 0.77, 95% CI 0.47–1.26, p = 0.30). The same multivariable Cox regression analyses were repeated after subgroup stratification according to the duration of CPR. Neither short nor long duration of CPR were found to be a risk factor for death-censored graft loss or death (Supplementary Table 4).

**TABLE 4:** Fixed effects of multivariable risk analysis of death-censored graft loss and mortality of kidney transplant recipients (n = 680 with 223 and 202 events, respectively).

Outcome	Death-censored graft loss		Mortality	
	Hazard ratio (95% CI)	p-value	Hazard ratio (95% CI)	p-value
Recipient age	1.02 (0.99–1.04)	0.16	1.08 (1.05–1.11)	*<0.001
Recipient sex: male vs. female	0.88 (0.66–1.17)	0.36	1.20 (0.87–1.67)	0.27
Recipient BMI	1.05 (1.02–1.09)	*0.003	1.03 (0.99–1.07)	0.12
Duration of dialysis	1.00 (1.00–1.01)	0.05	1.01 (1.00–1.01)	0.05
Recipient diabetes: yes vs. no	1.49 (1.07–2.07)	*0.02	1.48 (1.04–2.11)	*0.03
Recipient arterial hypertension: yes vs. no	0.74 (0.50–1.10)	0.14	1.12 (0.65–1.93)	0.69
Highest PRA	1.00 (0.99–1.01)	0.67	1.00 (0.99–1.01)	0.55
Number of HLA mismatches	1.07 (0.96–1.19)	0.24	0.93 (0.83–1.04)	0.22
Number of KTs	1.54 (0.91–2.58)	0.11	1.49 (0.84–2.64)	0.18
Cold ischemia time	1.01 (0.98–1.04)	0.64	1.03 (1.00–1.07)	*0.04
Donor age	1.02 (1.00–1.05)	0.10	1.01 (0.98–1.04)	0.54
Donor sex: male vs. female	1.00 (0.76–1.32)	1.00	0.75 (0.56–1.01)	0.06
Donor BMI	1.03 (0.99–1.07)	0.12	1.00 (0.96–1.05)	0.84
Donor cause of death cerebral infarction: yes vs. no	1.30 (0.81–2.07)	0.28	0.77 (0.51–1.15)	0.19
Donor arterial hypertension: yes vs. no	1.21 (0.89–1.64)	0.23	1.41 (0.99–2.03)	0.06
Donor diabetes: yes vs. no	0.84 (0.56–1.25)	0.39	1.09 (0.74–1.63)	0.66
Donor CPR: yes vs. no	0.76 (0.48–1.21)	0.25	0.77 (0.47–1.26)	0.30

BMI, body mass index; CI, confidence interval; CPR, cardiopulmonary resuscitation; HLA, human leucocyte antigen; KTs; kidney transplantations; PRA, panel reactive antigen. * if p < 0.05.

## Discussion

Even when performed optimally, CPR results in global hypoperfusion and ischemia. In turn, successful resuscitation is frequently accompanied by a multisystem injury and a systemic inflammatory response resembling severe sepsis [14, 15]. This response constitutes an additional insult to the inevitable ischemia-reperfusion injury intrinsic to organ transplantation. Increased susceptibility of organs from elderly donors has been postulated, and grafts from elderly donors with preceding CPR have therefore been subjected to heightened concerns regarding organ acceptance [16, 17].

Prior studies on the impact of CPR in KT had almost exclusively focused on younger donors (aged 18–50 years) and had observed no negative effect of a history of CPR on long-term allograft survival and function [5, 6]. In response to the ongoing organ shortage and the demographic change in our society, organ offers from far elder donors with a history of CPR have become common place. However, so far information on the outcome of such donors was very scarce. This provided the rationale for our study.

Our results demonstrate comparable allograft function as well as comparable death-censored graft survival and patient survival for KTs from ≥65-year-old DBD donors with and without a history of CPR. The incidence of BPAR was also not different. Stratification according to duration of CPR did not change the results–also for the long CPR group in which CPR duration was ≥20 min.

While these results are encouraging, we feel it is necessary to point out several selection criteria among the CPR donor group that we believe to be of high importance for the positive results of our study. First, all KTs assessed in this study were from DBD donors as DCD is prohibited in Germany. This is important as for DCD donors, worse allograft kidney function has been demonstrated also for younger CPR donors in some previous studies [18, 19]. Second, regarding CPR itself, median duration was fairly short (20 min), and the large majority of CPR donors had been resuscitated for ≤30 min (80%). While we did not observe a negative effect of CPR on allograft survival and function in the long CPR group, it is our belief that a CPR duration of >30 min in a ≥65-year-old DBD donor requires a very close scrutiny of the additional donor information before acceptance. This leads us to the third selection criteria. In our view, CPR as a donor characteristic cannot be regarded in isolation. Instead, we feel it is crucial to assess for CPR-associated organ damage (in the setting of KT especially severity of AKI and residual diuresis). In this regard, it is important to point out that even though the incidence of AKI was fairly high among CPR donors in our study (45.6%), most donors (84%) had only stage I AKI and none were oliguric, let alone anuric or on hemodialysis. Consequently, the median diuresis in the last 24 h prior to organ recovery was 3.0 L among CPR donors. Of note, we recently assessed the impact of donor AKI in ≥65-year-old DBD donors and found no difference in allograft survival and function for KTs from (primarily stage I) AKI donors with preserved diuresis [20]. Next to these CPR-associated aspects, we also consider CIT, donor comorbidities (especially arterial hypertension/diabetes) and donor age carefully in the selection process. Concerning these aspects, CIT in our study was kept short (median 10.2 h for CPR donors) in order to minimize the magnitude of ischemia-reperfusion injury (see section below) [4]. Also, CPR donors were slightly but significantly younger than no-CPR donors (70 years vs. 72 years) and almost all CPR donors were <80 years old (97%). We believe that these factors represent important donor selection criteria among ≥65-year-old DBD donors with a history of CPR that should be taken into account when considering a kidney offer of this kind. Therefore, our results do not imply that every ≥65-year-old CPR donor is a suitable candidate for KT. It has to be stressed that every organ offer needs to be assessed on a case-by-case basis after assessing the aforementioned factors as well as all other available donor information.

Another important aspect is recipient selection. The majority of kidneys in this study (>80%) were allocated through the Eurotransplant Senior Program (ESP). Within the ESP, kidneys from ≥65-year-old donors are allocated to ≥65-year-old recipients preferentially in the same geographical region and without HLA-matching [21, 22]. Therefore, recipients and donors were of comparable age (median donor age: 70 years in the CPR group and 72 years in the no-CPR group; median recipient age: 67 years in the CPR group and 67 years in the no-CPR group). The regional allocation also explains the short CIT in both groups which is a typical characteristic of the ESP [21, 23, 24]. Hence, we would suggest to offer a kidney from a ≥65-year-old CPR donor preferentially to a similarly aged recipient. Of note, the regional allocation is also the reason why, in 19.5% of donors, we were offered both kidneys from the same donor and subsequently transplanted them into two separate recipients. In turn, the number of KTs (n = 680) was higher than the number of kidney donors (n = 569).

There have been previous studies on the effect of donor CPR on kidney allograft survival [5, 6]. The most recent meta-analysis by Sandroni et al. showed no difference in allograft survival between 950 DBDs with a history of CPR and 12,719 DBDs without a history of CPR after 1 year and actually even a small survival advantage at the longest follow-up (even though this time point was not defined more clearly; p = 0.04) [6]. However, virtually all studies included in the meta-analysis had focused on younger donors (aged primarily 18–50 years) which is why the median donor age of 70 years in our CPR group was 20 years higher than in any other study from the meta-analysis. Yet, nowadays, kidney transplant physicians in countries with low organ donation rates (such as Germany) regularly receive kidney offers from ≥65-year-old DBD donors with a history of CPR which highlights the knowledge gap about this vulnerable donor group. The only other publication specifically analyzing the effect of CPR in ≥65-year-old DBD donors came from our own group [7]. However, this previous paper had several shortcomings as it was a single-center analysis with a considerably smaller sample size (n = 185) and covering a longer time span (1999–2019) in which mycophenolate-based immunosuppression and induction therapy was not yet standard of care. In contrast, the current data set was derived from three transplant centers comprising more than three times the previous sample size (n = 680) and covering a different time period (2006–2023), in which mycophenolate-based immunosuppression and induction therapy had become standard of care. These facts make the current paper more reliable and more relevant to everyday practice.

Of note, we also report no significant difference in the incidence of DGF and PNF between the CPR and no-CPR groups (27.2% vs. 33.1% and 9.9% vs. 12.2%, respectively). These numbers are comparable to previously published data on ESP donors and ECDs aged ≥65 years [21, 25, 26]. The aforementioned meta-analysis also showed no significant difference in the incidence of DGF between the CPR and the no-CPR groups. Mechanistically, one could speculate that successful CPR allows the restoration of physiological organ function already during the stabilization phase in the intensive care unit, when considerations regarding organ donation are being undertaken. This may in turn reduce the incidence of DGF. Ultimately, we can only speculate about the reasons.

It is important to realize that the results of this study may have been influenced by donor selection bias because we can only report on the results of those kidney organ offers that had been accepted for KT in the first place. Offers that had been declined were not available for outcome analysis.

In summary, we demonstrate comparable 5-year death-censored graft survival as well as up to 5-year allograft function between KT from ≥65-year-old DBD donors with and without a history of CPR. These encouraging results indicate that KT from ≥65-year-old CPR donors is a viable option especially when the donors have no or only stage I AKI with preserved diuresis and both the duration of CPR as well as the expected CIT are short (ideally <30 min and <12 h, respectively). These results may help to further enhance the pool of suitable elderly kidney donors.

## Data Availability

The raw data supporting the conclusions of this article will be made available by the authors, without undue reservation.
