# Supplementary material for: Cardiopulmonary resuscitation in donation after brain death donors aged ≥65 years: impact on outcomes after kidney transplantation – a multi-center study
Source: Transpl Int. 2026 May 22;39:16028. doi: 10.3389/ti.2026.16028 (PMC13236627; doi:10.3389/ti.2026.16028)
Supplement: Supplementary file 1 [file Supplementaryfile1.docx]

**Supplementary materials**

**Supplementary Table 1.** Donor characteristics of kidney transplants, stratified according to the duration of

donor CPR

| **Characteristic** | **Short CPR Group (n=35)** | **Long CPR Group**  **(n=33)** | **No-CPR Group (n=501)** | **p-value** |
| --- | --- | --- | --- | --- |
| Age (years), median (IQR) | 72 (67.5, 74.5) | 70 (68.0, 71.0) | 72 (68.0, 77.0) | 0.04* |
| Male sex | 12 (34.3%) | 16 (48.5%) | 237 (47.3%) | 0.31 |
| BMI, median (IQR) | 26.9 (24.8, 28.7) | 26.9 (24.6, 29.3) | 26.2 (24.2, 28.7) | 0.24 |
| Arterial hypertension | 26 (76.5%) | 21 (65.6%) | 295 (63.3%) | 0.28 |
| Diabetes | 3 (9.4%) | 11 (36.7%) | 77 (17.0%) | 0.02* |
| Smoking | 4 (11.4%) | 16 (53.3%) | 108 (24.3%) | 0.001* |
| Cause of death |  |  |  | <0.001* |
| Stroke  Intracranial bleeding  Trauma  Other (e.g., anoxic brain damage) | 6 (17.1%)  19 (54.3%)  1 (3.0%)  9 (25.7%) | 3 (9.1%)  9 (27.3%)  1 (2.9%)  20 (60.6%) | 62 (12.4%)  295 (58.9%)  58 (11.6%)  86 (17.2%) |  |
| eGFR (ml/min/1.73 m²) |  |  |  |  |
| On admission, median (IQR)  Lowest, median (IQR)  Final, median (IQR) | 70.0 (58.4, 86.8)  61.0 (43.2, 76.8)  69.1 (52.7, 91.6) | 66.0 (50.0, 74.3)  59.0 (37.0, 68.1)  66.0 (42.6, 89.9) | 84.4 (68.3, 92.0)  71.3 (57.5, 87.4)  81.0 (62.3, 93.0) | <0.001*  <0.001*  0.01* |
| Diuresis in last 24 hours prior to organ donation (litres), median (IQR) | 3.7 (2.2, 6.2) | 2.7 (1.5, 3.3) | 3.6 (2.4, 4.8) | 0.005* |
| AKI stage |  |  |  | 0.003* |
| No AKI  Stage 1  Stage 2+3 | 21 (60.0%)  12 (34.3%)  2 (5.8%) | 16 (48.5%)  14 (42.4%)  3 (9.1%) | 379 (76.6%)  100 (20.2%)  16 (3.2%) |  |
| Ongoing AKI at kidney recovery | 6 (40.0%) | 7 (38.9%) | 80 (64.0%) | 0.04* |
| Time from admission to organ procurement (days), median (IQR) | 4 (2, 5) | 4 (2, 5) | 3 (2, 5) | 0.73 |
| KDPI, median (IQR) | 75.0 (60.0, 88.0) | 81.0 (61.0, 89.0) | 74.5 (62.0, 88.0) | 0.94 |
| Duration of CPR, median (IQR) | 12.5 (1, 18) | 30 (20, 60) |  |  |
| Out-of-hospital cardiac arrest | 28 (80.0%) | 30 (90.9%) |  |  |

Percentages are based on the number of available cases for each parameter. Kruskal–Wallis test was used for comparison of numeric variables and Fisher´s exact test (i.e., Chi2-test) was used for comparison of categorical variables. AKI, acute kidney injury; BMI, body mass index; CPR, cardiopulmonary resuscitation; CVA, cerebrovascular accident; IQR, interquartile range; * if p <0.05.

**Supplementary Table 2.** Recipient and transplant characteristics of kidney transplants, stratified according to the duration of donor CPR

| **Characteristic** | **Short CPR Group**  **(n=39)** | **Long CPR Group**  **(n=42)** | **No-CPR Group**  **(n=599)** | **p-value** |
| --- | --- | --- | --- | --- |
| Age (years), median (IQR) | 67.0 (65.0, 69.0) | 66.0 (65.0, 68.8) | 67.0 (65.0, 70.0) | 0.84 |
| Male sex | 28 (71.8%) | 28 (66.7%) | 404 (67.4%) | 0.88 |
| BMI, median (IQR) | 26.4 (24.7, 29.2) | 27.3 (24.1, 29.2) | 25.7 (23.6, 29.1) | 0.33 |
| Duration of dialysis (months), median (IQR) | 43 (33, 66) | 56 (36, 79) | 52 (31, 75) | 0.62 |
| RRT |  |  |  | 0.54 |
| Hemodialysis  Peritoneal dialysis | 33 (84.6%)  6 (15.4%) | 36 (85.7%)  6 (14.3%) | 530 (88.8%)  67 (11.2%) |  |
| Underlying renal disease |  |  |  | 0.46 |
| Diabetic nephropathy  Hypertensive nephropathy  Polycystic kidney disease  Glomerulonephritis  Other  Unknown | 6 (15.4%)  5 (12.8%)  7 (17.9%)  11 (28.2%)  4 (10.3%)  6 (15.4%) | 4 (9.5%)  9 (21.4%)  4 (9.5%)  14 (33.3%)  8 (19.0%)  3 (7.1%) | 72 (12.0%)  59 (9.9%)  83 (13.9%)  180 (30.1%)  121 (20.2%)  83 (13.9%) |  |
| Arterial hypertension | 28 (87.5%) | 37 (90.2%) | 445 (87.3%) | 0.93 |
| Diabetes | 11 (34.4%) | 6 (14.6%) | 119 (23.3%) | 0.15 |
| Highest PRA |  |  |  | 0.009* |
| 0%  >0 – ≤20%  >20% | 27 (69.2%)  5 (12.8%)  7 (17.9%) | 25 (59.5%)  8 (19.0%)  9 (21.4%) | 337 (57.3%)  189 (32.1%)  62 (10.5%) |  |
| Second/third kidney transplant | 3 (7.7%) | 8 (19.1%) | 40 (6.7%) | 0.04* |
| HLA mismatches |  |  |  | 0.50 |
| 0  1–2  3–4  5–6 | 1 (2.6%)  5 (12.8%)  18 (46.2%)  15 (38.5%) | 2 (4.8%)  7 (16.7%)  18 (42.9%)  15 (35.7%) | 10 (1.7%)  64 (10.8%)  283 (47.6%)  238 (40.0%) |  |
| Cold ischemia time (hours), median (IQR) | 10.0 (6.9, 13.0) | 11.4 (8.4, 13.9) | 11.2 (8.1, 15.7) | 0.31 |
| Immunosuppression |  |  |  |  |
| IL2-RA/ATG/none  Tac/CsA/other  MMF/Aza  Corticosteroids | 87.2%/10.3%/2.6%  79.5%/20.5%/0.0%  100%/0.0%  97.4% | 81.0%/19.0%/0.0%  61.9%/38.1%/0.0%  100%/0.0%  100% | 88.7%/7.5%/3.8%  56.0%/43.1%/0.8%  99.7%/0.3%  98.0% | 0.10  0.06  1  0.65 |
| Year of transplantation, median (IQR) | 2015 (2010, 2018) | 2016 (2012, 2020) | 2014 (2010, 2018) | 0.15 |

Percentages are based on the number of available cases for each parameter. Kruskal–Wallis test was used for comparison of numeric variables and Fisher´s exact test (i.e., Chi2-test) was used for comparison of categorical variables. AKI, acute kidney injury; IQR, interquartile range; BMI, body mass index; RRT, renal replacement therapy; PRA, panel reactive antigen; HLA, human leukocyte antigen; SD, standard deviation, IL2-RA, interleukin 2-receptor antibody; ATG, antithymocyte globulin; Tac, tacrolimus; CsA, Ciclosporine A; MMF, mycophenolate mofetil; Aza, azathioprine, ESP, Eurotransplant Senior Program.

**Supplementary Table 3.** Short and long-term outcomes of kidney transplants stratified according to the duration of donor CPR

| **Characteristic** | **Short CPR Group (n=39)** | **Long CPR Group (n=42)** | **No-CPR Group (n=599)** | **p-value** |
| --- | --- | --- | --- | --- |
| DGF | 10 (25.6%) | 12 (28.6%) | 197 (33.1%) | 0.38 |
| PNF | 6 (15.4%) | 2 (4.8%) | 73 (12.2%) | 0.29 |
| Recipient eGFR (ml/min/1.73 m²), median (IQR) |  |  |  |  |
| 3 months after transplant  1 year after transplant  3 years after transplant  5 years after transplant | 30.4 (22.4, 43.8)  33.5 (27.2, 40.3)  37.6 (26.3, 52.4)  33.1 (30.3, 38.5) | 38.0 (25.1, 48.0)  33.2 (25.7, 48.1)  38.5 (29.5, 49.0)  40.4 (34.7, 49.8) | 34.0 (24.3, 43.8)  35.0 (26.9, 45.0)  35.0 (27.0, 47.4)  37.3 (26.8, 49.2) | 0.56  0.81  0.72  0.63 |
| BPAR in first three years after KT | 13 (36.1%) | 17 (44.7%) | 230 (41.8%) | 0.25 |
| Death-censored graft survival |  |  |  | 0.18 |
| 1-year  3-year  5-year | 74.1%  71.4%  67.6% | 89.9%  89.9%  78.8% | 80.0%  72.1%  66.0% |  |
| Patient survival |  |  |  | 0.85 |
| 1-year  3-year  5-year | 89.5%  81.0%  81.0% | 90.2%  81.0%  76.0% | 90.2%  80.2%  73.5% |  |
| Follow-up time (months), median (IQR) | 44 (23, 72) | 36.0 (16, 59) | 41 (23, 81) | 0.4 |

Percentages are based on the number of available cases for each parameter. Kruskal–Wallis test was used for comparison of numeric variables and Fisher´s exact test (i.e., Chi2-test) was used for comparison of categorical variables. Death-censored graft survival and patient survival rates were calculated using the Kaplan−Meier method; AKI, acute kidney injury; BPAR, biopsy proven acute rejection; DGF, delayed graft function; PNF, primary non-function; IQR, interquartile range.

**Supplementary Table 4.** Fixed effects of multivariable risk analysis of death-censored graft loss and mortality of kidney transplant recipients with a history of no-, short- and long donor CPR (n=680 with 223 and 202 events, respectively)

| **Outcome** | **Death-censored graft loss** | | **Mortality** | |
| --- | --- | --- | --- | --- |
|  | **Hazard ratio (95% CI)** | **p-value** | **Hazard ratio (95% CI)** | **p-value** |
| Recipient age | 1.02 (0.99–1.04) | 0.14 | 1.08 (1.05–1.11) | *<0.001 |
| Recipient sex: male vs. female | 0.85 (0.64–1.16) | 0.27 | 1.21 (0.87–1.68) | 0.26 |
| Recipient BMI | 1.05 (1.02–1.10) | *0.003 | 1.03 (0.99–1.07) | 0.10 |
| Duration of dialysis | 1.01 (1.00–1.01) | *0.03 | 1.01 (1.00–1.01) | 0.04 |
| Recipient diabetes: yes vs. no | 1.50 (1.07–2.10) | *0.02 | 1.48 (1.02–2.15) | 0.04 |
| Recipient arterial hypertension: yes vs. no | 0.76 (0.49–1.16) | 0.20 | 1.12 (0.66–1.91) | 0.68 |
| Highest PRA | 1.00 (0.99–1.01) | 0.95 | 1.00 (1.00–1.01) | 0.51 |
| Number of HLA mismatches | 1.07 (0.96–1.19) | 0.24 | 0.93 (0.83–1.04) | 0.20 |
| Number of KTs | 1.57 (0.88–2.60) | 0.09 | 1.58 (0.90–2.78) | 0.11 |
| Cold ischemia time | 1.01 (0.98–1.04) | 0.54 | 1.03 (0.99–1.06) | 0.06 |
| Donor age | 1.02 (0.99–1.05) | 0.09 | 1.01 (0.98–1.04) | 0.53 |
| Donor sex: male vs. female | 1.00 (0.76–1.32) | 0.98 | 0.75 (0.55–1.00) | 0.05 |
| Donor BMI | 1.03 (0.99–1.08) | 0.10 | 1.00 (0.96–1.05) | 0.87 |
| Donor cause of death cerebral infarction: yes vs. no | 1.41 (0.88–2.28) | 0.16 | 0.73 (0.49–1.10) | 0.13 |
| Donor arterial hypertension: yes vs. no | 1.24 (0.91–1.70) | 0.18 | 1.35 (0.96–1.90) | 0.09 |
| Donor diabetes: yes vs. no | 0.87 (0.57–1.33) | 0.51 | 1.11 (0.74–1.67) | 0.63 |
| Donor CPR short: yes vs. no | 1.14 (0.65–1.97) | 0.63 | 0.69 (0.36–1.34) | 0.28 |
| Donor CPR long: yes vs. no | 0.45 (0.25–1.04) | 0.09 | 0.90 (0.46–1.78) | 0.76 |

AKI, acute kidney injury; BMI, body mass index; CI, confidence interval; CVA, cerebrovascular accident; HLA, human leucocyte antigen; KTs; kidney transplantations; PRA, panel reactive antigen. * if p < 0.05.

**Capsule Sentence Summary**

Kidney transplantation from ≥65-year-old DBD donors with and without CPR yields comparable 5-year graft survival and function. Important donor selection criteria include CPR duration (ideally <30 minutes), no or only stage I AKI and cold ischemia time (ideally <12 hours).
